# Primary Liver Abscess Caused by One Clone of *Klebsiella pneumoniae* with Two Colonial Morphotypes and Resistotypes

**DOI:** 10.3201/eid0801.010167

**Published:** 2002-01

**Authors:** Po-Ren Hsueh, Juinn-Jong Wu, Lee-Jene Teng, Yee-Chun Chen, Pan-Chyr Yang, Shen-Wu Ho, Kwen-Tay Luh

**Affiliations:** * National Taiwan University Hospital, Taipei, Taiwan; † National Cheng-Kung University Medical College, Tainan, Taiwan; ‡ National Taiwan University College of Medicine, Taipei, Taiwan

**Keywords:** *Klebsiella pneumoniae*, liver abscess, diabetes

## Abstract

Two diabetic patients with primary liver abscess, who initially responded unsatisfactorily to intravenous ceftriaxone or cefoxitin treatment and had abscess drainage, were found to be infected with a single clone of *Klebsiella pneumoniae* with two different colonial morphotypes and resistotypes. Primary liver abscess caused by second-generation cephalosporin-resistant *K. pneumoniae* strains may be an emerging problem in Taiwan.

Primary liver abscess caused by a single pathogen, *Klebsiella pneumoniae*, has long been an important infectious complication in diabetic patients in Taiwan (*1*,*2*). All *K. pneumoniae* strains causing primary liver abscess have a unique antimicrobial susceptibility pattern (resistant to ampicillin, ticarcillin, and carbenicillin but susceptible to other antibiotics including all cephalosporins and aminoglycosides) (*1*–*4*). Although multidrug-resistant strains of *K. pneumoniae,* whether community acquired or nosocomial, are not uncommon in Taiwan and other countries, these isolates had never been reported as the cause of primary liver abscess (*5*,*6*).

## Case Summaries

**Figure 1:**
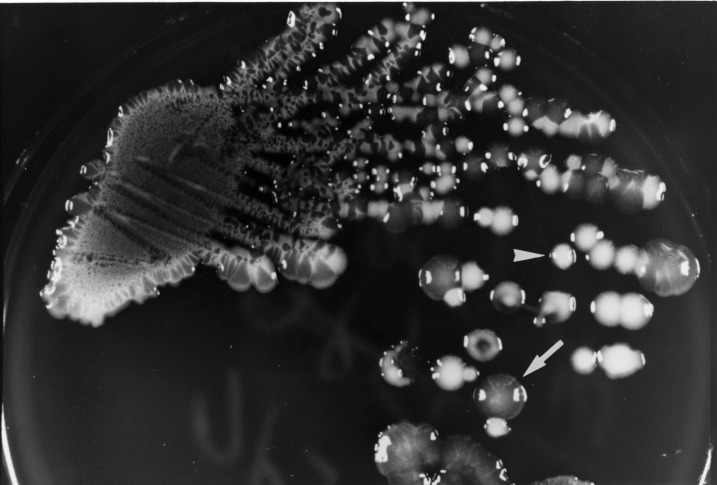
Colonial morphology of *Klebsiella pneumoniae* grown on a primary isolation plate (trypticase soy agar supplemented with 5% sheep blood) from the abscess aspirate of patient A after 24 hours of incubation. The arrow shows a mucoid opaque colony (isolate A1). The arrowhead shows a nonmucoid white colony (isolate A2).

A 42-year-old man (patient A) was admitted on July 31, 2000, for a fever of 2 weeks’ duration. He had had diabetes mellitus for 20 years and alcoholism for >10 years. Physical examination and abdominal echography showed hepatomegaly and a huge abscess (12 cm x 10 cm x 8 cm) over the right lobe of the liver. Laboratory tests showed leukocytosis (14,600/μL) with a left shift. After blood cultures, ceftriaxone (2 g every 12 hours) was given. A “pigtail” catheter was inserted for continuous drainage on the 5th hospital day. Abscess aspirate culture yielded *K. pneumoniae* with two colonial morphotypes (isolates on trypticase soy agar plates supplemented with 5% sheep blood [BBL Microbiology Systems, Cockeysville, MD] after 24 hours of incubation in ambient air) (Figure 1) and two resistotypes (by the routine disk diffusion method). One (isolate A1) had mucoid, opaque colonies and was resistant to ampicillin but susceptible to cefazolin and cefoxitin, and the other (isolate A2) had nonmucoid, white colonies and was resistant to ampicillin, cefazolin, and cefoxitin. Both isolates were susceptible to amoxicillin-clavulanate and cefotaxime. Blood cultures were negative. Because of persistent fever, the antibiotic was changed to imipenem (500 mg every 6 hours) on the 11th hospital day. Fever subsided 3 days after imipenem administration was begun. Imipenem was continued for a total of 24 days, followed by ciprofloxacin (750 mg every 12 hours) for 3 weeks. A follow-up echography 4 months after antibiotic treatment ended showed that the abscess had disappeared.

A 66-year-old man (patient B) with diabetes mellitus was admitted on August 4, 2000, with fever and hiccups of 4 days’ duration. Physical examination was unremarkable. Laboratory tests showed elevated levels of alkaline phosphatase (585 U/L; reference range 66-220 U/L) and gamma-glutamyltranspeptidase (301 U/L; reference range <52 U/L) but normal levels of aspartate and alanine aminotransferases. Abdominal echography and computed tomography revealed an abscess (6 cm x 4 cm x 4 cm) over the right lobe of the liver and a gallbladder stone. Cefoxitin (2 g every 8 hours) was given intravenously. The abscess was aspirated on the 4th day after hospitalization. Two isolates (isolates B1 and B2) of *K. pneumoniae* with different colonial morphotypes and resistotypes (as in isolates A1 and A2, respectively, from patient A) were found in one aspirate culture, and two isolates (isolates B3 and B4) of the same species with different phenotypes (as in isolates B1 and B2, respectively) were recovered from one set of blood cultures, performed at admission. Cefoxitin was discontinued, and cefotaxime (2 g every 6 hours) was administered. His fever resolved 2 days after cefotaxime administration was begun. The patient received cefotaxime for 15 days, followed by oral cefixime (200 mg every 12 hours) for 7 weeks. Follow-up echography showed the abscess had disappeared.

In vitro susceptibilities of 14 antimicrobial agents for these isolates were determined by using the standard agar dilution method (*7*). Biotyping of thee isolates was performed with the API ID32 GN system (bioMerieux, Marcy I'Etoile, France). Random amplified polymorphic DNA (RAPD) patterns of the six isolates were determined by means of arbitrarily primed polymerase chain reaction, as described in our previous report (*8*). A total of four primers were used: M13 (5'-TTATGTAAAACGACGGCCAGT-3'), OPH2, OPA3, and OPA9 (Operon Technologies, Inc., Alameda, CA). Pulsotypes of these isolates were determined by pulsed-field gel electrophoresis; plasmid analysis and conjugative experiment were performed as described (*3*). For comparisons, molecular typing of an additional two *K. pneumoniae* isolates recovered from two patients seen at our hospital in 1999 were performed simultaneously.

The cefoxitin- and cefuroxime-resistant isolates (isolates A2, B2, and B4) showed two- to four-fold higher MICs of cefotaxime and ceftriaxone (MICs 0.5-1 μg/mL), ceftazidime (MICs 1 μg/mL), flomoxef (MICs 0.5-2 μg/mL), ticarcillin-clavulanate (MICs 8 μg/mL), piperacillin-tazobactam (MICs 4-8 μg/mL), cefepime (MICs 0.5-1 μg/mL), cefpirome (MICs 0.12-1 μg/mL), imipenem (MICs 2 μg/mL), and meropenem (MICs 0.12 μg/mL) than those of the cefoxitin-susceptible isolates (isolates A1, B1, and B3). All isolates were susceptible to the above antibiotics and aminoglycosides (gentamicin and amikacin).

**Figure 2:**
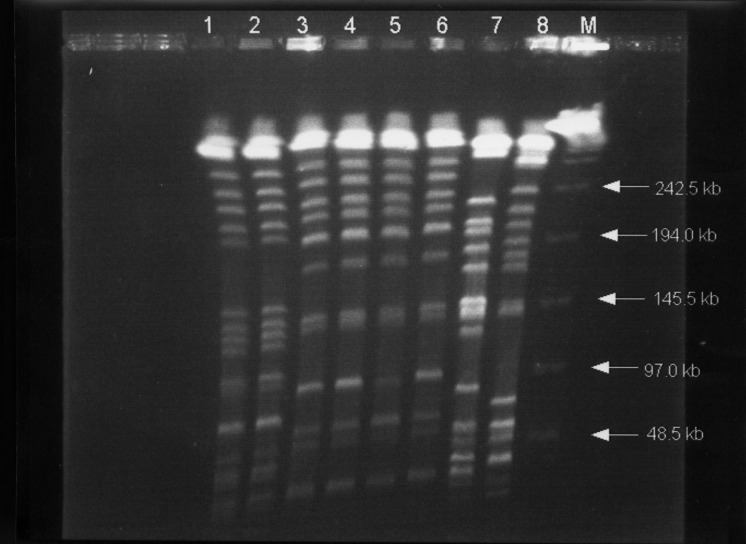
Pulsed-field gel electrophoresis profiles of XbaI-digested genomic DNAs from eight *Klebsiella pneumoniae* isolates. Lanes 1 and 2, profiles for isolates A1 and A2 (from patient A); lines 3 to 6, profiles for isolates B1 to B4 (from patient B), respectively; and lanes 7 and 8, isolates of *K. pneumoniae* from two other patients used as control strains. Lane M, bacteriophage lamdba DNA concatemers (GibcoBRL, Gaithersburg, MD)

As shown in the Table, the identity of biotypes, RAPD patterns with four primers, and pulsotypes (Figure 2) was clearly demonstrated for the isolates with two different phenotypes from each patient, suggesting that they belonged to a single clone in each patient (clone 1, isolates A1 and A2; clone 2, isolates B1 to B4). Different clones isolated from two patients seen within 1 week indicate that no outbreak occurred. Isolates A1 and A2 both had two plasmids (50 kb and 5 kb). Only one plasmid (30 kb) was found in each of the four isolates (B1 to B4) of patient 2. All these plasmids cannot transfer to *Escherichia coli* C600.

**Table:** Clinical characteristics of two diabetic patients with liver abscess and microbiologic characteristics of *Klebsiella pneumoniae* isolates recovered from them

Patient designation (age, yr/gender)	Antibiotic treatment (days)	Sources of isolate/ designation	Characteristics of isolates						
			Colonial morphotype	Res Resistotype (MIC, mg/mL)^a^				Bio Biotype	RAPD pattern
				AMP	CZ	FOX (CXM)	CTX (CRO)		
A (42/M)	Ceftriaxone (10)	Abscess fluid							
	Imipenem (24)	A1	Mucoid	64	4	16	0.12	I	a/1
	Ciprofloxacin (21)	A2	Nonmucoid	128	32	128	1	I	a/1
B (66/M)	Cefoxitin (10)	Abscess fluid							
	Cefotaxime (15	B1	Mucoid	32	2	4	0.06	I	b/2
	Cefixime (35)	B2	Nonmucoid	128	32	128	0.5	I	b/2
		Blood samples							
		B3	Mucoid	32	2	4	0.03	I	b/2
		B4	Nonmucoid	128	64	256	0.5	I	b/2

^a^AMP, ampicillin; CZ, cefazolin; FOX, cefoxitin, CXM, cefuroxime, CTX, cefotaxime, CRO, ceftriaxone.

RAPD = random amplified polymorphic DNA.

## Conclusions

Although infection caused by a single clone of one bacterial species that simultaneously possessed two obviously different colonial morphotypes has been noted previously (*8*), infection caused by a single clone of *K pneumoniae* exhibiting two discrete colonial morphotypes and resistotypes has never been reported. Primary liver abscess due to *K. pneumoniae* having resistance to second-generation cephalosporins (MIC up to 128 μg/mL for isolate B2 and 256 μg/mL for isolate B4 in patient B) and higher MICs of third-generation cephalosporins (MICs up to 1 μg/mL for isolate A2 in patient A) has also not been previously reported. We believe that this decreased in vitro susceptibility contributed to the unsatisfactory response to treatment with these agents.

The genetic basis for the resistance to cephalosporins and for the mucoid material synthesis by some gram-negative bacteria may be chromosomally determined or dependent on the acquisition of a specific plasmid or phage (*5*,*9*,*10*). The resistant and mucoid material synthesis may also be regulated by the environment in which the bacteria grow (*5*,*9*,*10*). The association of a specific plasmid with the presence of mucoid phenotype was not found in our isolates because both mucoid and nonmucoid strains of the two clones of *K. pneumoniae* had identical plasmid profiles. The mechanisms of the coexistence of two phenotypically distinct isolates within a single clone *K. pneumoniae* in abscess fluid, blood, or both should be investigated. The mechanism of cefoxitin resistance in our isolates was unclear because of the failure of transferability of the plasmids existing in our isolates to the susceptible recipient. Whether this resistance was chromosome mediated or caused by other mechanisms needs further investigations.

These two cases suggest that primary liver abscess caused by multiresistant strains of *K. pneumoniae* in diabetic patients may be an emerging problem in Taiwan. The belief that only strains of *K. pneumoniae* that are susceptible to cephalosporins cause primary liver abscess has now been disproved.
